# Family Medicine in Gulf Cooperation Council Countries: Perspectives, Directions, and Future Opportunities; A Narrative Review

**DOI:** 10.3390/healthcare14111514

**Published:** 2026-05-29

**Authors:** Asma Said Hamed Al Shidhani, Maisa Hamed Al Kiyumi, Buthaina Ahmed Al Zaabi, Badriya Saleh Al Farsi, Faisal A. Alnaser, Abdulaziz Al Mahrezi

**Affiliations:** 1 Family Medicine and Public Health, Sultan Qaboos University Hospital, Sultan Qaboos University, Muscat 123, Oman; asma.shidhani@hotmail.com (A.S.H.A.S.); abdulaziz@squ.edu.om (A.A.M.); 2 Primary Health Care, Ministry of Health, Muscat 100, Oman; buthaina.alzaabi@moh.gov.om; 3 The Medical City for Military and Security Services (MCSS), Muscat 112, Oman; dr.badriyaalfarsi@gmail.com; 4 Honorary Faculty, Imperial College London, London SW7 2AZ, UK; falatf@gmail.com

**Keywords:** family practice, primary health care, Gulf Cooperation Council, health systems, health services accessibility, preventive health services, telemedicine, medical education, delivery of health care

## Abstract

Family medicine has attracted increasing policy and institutional support across the Gulf Cooperation Council (GCC) countries through health system reform, expansion of the healthcare workforce, and sustained public investment. Nevertheless, important challenges continue to affect the strength of primary healthcare systems, access to care, and the management of non-communicable diseases. The aim of the narrative review is to identify future trends, directions, perspectives, and opportunities that can strengthen implementation of family medicine across GCC countries and improve healthcare delivery. This review is based on a structured search of major databases such as PubMed, Scopus, and Google Scholar. The focus was evaluation of literature associated with family medicine and primary healthcare development in GCC countries. Regional priorities now include improving medical education and training, expanding the family medicine workforce, strengthening links with communities, promoting more equitable access to healthcare, and managing treatment costs through workforce development and digital health initiatives. Family medicine practice across the GCC is being supported increasingly by electronic health records, telemedicine, and interprofessional education. Policy directions in the region also suggest growing interest in value-based research, international collaboration, multidisciplinary care, and innovation in healthcare delivery. The future of development of family medicine in the GCC will depend on better integration of digital health, more effective use of data in planning and policy, continued investment in training, and broader adoption of patient-centred models of care. In general, strengthening family medicine through sustained investment in workforce development, primary healthcare infrastructure, research capacity, and digital health integration is essential for achieving resilient, equitable, and patient-centered healthcare systems across the GCC.

## 1. Introduction

Healthcare systems worldwide are being shaped increasingly by developments in family medicine [1]. Over the past 25 years, the expansion of healthcare systems across the Gulf Cooperation Council (GCC) countries has largely reflected changing patterns of patient need [2]. In parallel, the healthcare workforce across the region has grown in response to increasingly diverse service demands [3].

Following the economic transformation associated with oil production, the disease profile in the GCC changed markedly as life expectancy and lifestyle patterns shifted. Non-communicable diseases and obesity-related conditions became major contributors to the regional burden of the disease, placing increasing pressure on healthcare systems. These changes required corresponding adjustments in resource allocation, workforce training, and strategic planning [4,5]. Poor health-related quality of life is also an important contributor to chronic disease, including cardiometabolic disorders [6]. The high prevalence of these conditions has been linked to more than 152,000 deaths, rising disability-adjusted life years, and an increased healthcare burden. Growing incidence rates of cancer and respiratory disease have added further financial pressure to health systems. At the same time, disparities in access to healthcare continue to weaken equity in treatment [7].

Across the GCC, the healthcare workforce is continuing to grow, with approximately 2483 new workers added each year. If this pattern continues, the total number of healthcare professionals is projected to reach 54,231 by 2029 [8]. Considerable variation remains evident across the region. In Abu Dhabi, for example, workforce density is nearly double that reported for the UAE overall, with 134.7 versus 50.4 nurses and 52.4 versus 23.2 physicians per 10,000 population. In Bahrain, government-administered primary care services operate 28 patient support centres [9]. In Oman, the healthcare workforce in 2019 was reported at 44 nurses and 21 physicians per 10,000 population [10]. In the Kingdom of Saudi Arabia, the total health workforce was estimated at 467,650, of whom 7% were pharmacists, 20% physicians, 26% allied health professionals, and 43% nurses [11]. In Qatar, the reported average numbers of dentists, nurses, and physicians were 1135.5, 13,118, and 4343.5, respectively [12]. In Kuwait, Ministry of Health reports recorded a workforce of more than 66,000, including physicians, nurses, pharmacists, technicians, and administrative personnel [13].

Even with this expansion, primary care systems across most GCC countries continue to face comparable pressures. A continuing shortage of family physicians and other primary care providers remains a major concern. In many parts of the region, small populations spread over large geographic areas have contributed to ongoing dependence on expatriate healthcare workers [14]. This can weaken system stability, as high staff turnover may interrupt continuity of care and increase the need for repeated in-service training. All GCC countries have established family medicine residency programmes [14]. However, current training capacity is still not sufficient to meet growing healthcare demands, largely because of low trainee enrolment, limited numbers of supervisors and accredited trainers, and restrictions in available training sites [15]. At the same time, GCC countries have shown increasing awareness of the need for more innovative and cost-conscious healthcare strategies built around family medicine [7]. International developments in family medicine also point to the importance of diversifying the workforce, strengthening residency training, supporting primary care scholarship, expanding value-based research, and developing workforce policy at the national level [16]. Worldwide, family physicians make up nearly 40% of the primary care physician workforce, although the reasons for the wide variation in their scope of practice across settings remain unclear [17].

The need to transform healthcare systems through stronger primary care interventions has therefore remained a priority across the GCC [14]. In response, several countries have introduced national health strategies, including Oman’s Health Vision 2040, Bahrain’s National Health Plan, and Saudi Arabia’s Vision 2030 [18]. The aim of this narrative review is to identify future trends, directions, perspectives, and opportunities that can be used to strengthen implementation of family medicine across GCC.

## 2. Methodology

This review involves a structured literature search from inception to September 2025. Electronic databases that were used include PubMed, Scopus, and Google Scholar. Search terms included combinations of: “family medicine,” “family practice,” “primary health care,” “general practice,” “Gulf Cooperation Council,” “GCC,” “Oman,” “Saudi Arabia,” “United Arab Emirates,” “Qatar,” “Kuwait,” “Bahrain,” “medical education,” “telemedicine,” “digital health,” “research,” and “primary care.” Studies selected had to be published in English and cover topics such as family medicine, primary healthcare systems, medical education, research, healthcare policy, or digital health relevant to GCC countries. There was a review of policy documents and reports from international organizations for information to support objectives. Resources that were selected had to address the relevance of the topic and contribute to understanding healthcare system strengthening, medical education, research, and public engagement in the region. Data from the articles was synthesized to determine key trends, challenges and opportunities. This approach was significant in developing a broad and interactive perspective of the topic.

## 3. Strengthening the Primary Healthcare Systems

### 3.1. Government Policy and Support Measures

The strengthening of primary healthcare in GCC countries depends not simply on the presence of national policies, but on the extent to which those policies are translated into practice. This calls for regular assessment of system performance, sustained financial investment, a capable and well-supported workforce, and legislative frameworks that facilitate service development [19]. In recent years, primary healthcare has moved closer to the center of health reform across GCC countries because of its recognized role in improving both service quality and patient outcomes [5,20]. In Saudi Arabia, Vision 2030 gives particular attention to prevention, health promotion, and community-based care. Bahrain’s National Health Plan and Oman’s Health Vision 2040 similarly treat primary healthcare as a key part of achieving universal health coverage [2,21,22,23]. These policy directions also point to greater interest in integrated care, digital health, and more decentralized forms of service delivery. Across the region, wider health coverage and a stronger place for family medicine remain recurring priorities in ongoing reform efforts [24]. There is also increasing support for care models that are person-centered, holistic, and multidisciplinary, while still drawing on relevant international experience [25]. The value of these reforms depends on continued review of healthcare costs, patient satisfaction, access to services, and other indicators of system performance. Indicators such as Patient-Oriented Evidence That Matters (POEMs) evaluate outcomes that directly affect patient care, quality of life, and clinical decision-making [25]. A summary of key national health strategies and their primary focus areas across GCC countries is presented in Table 1 to provide a comparative overview of policy directions.

### 3.2. Regional and International Partnerships

Regional and international partnerships have also taken on a more important role in efforts to strengthen primary healthcare across the GCC [5]. International partnerships contribute to the advancement of evidence-based medicine. They promote exchange of scientific knowledge, foster collaborative research, and support capacity building in healthcare systems. Collaborating with international organizations and academic institutions is significant for GCC countries. It allows the countries to access high-quality evidence, adopt best practices, and adapt global guidelines to local contexts. These partnerships promote development of research infrastructure and support evidence-informed policy-making to strengthen primary healthcare systems and improve patient outcomes [5]. Current initiatives led by the World Health Organization Eastern Mediterranean Regional Office aim to help member states refine and standardize primary healthcare approaches [26]. At the same time, the continued development of family medicine depends on stronger evidence-informed decision-making and more consistent use of scientific data in health policy [27]. An example is the World Health Organization Eastern Mediterranean Regional Office implementing the Primary Health Care Measurement and Improvement (PHCMI) initiative. The program helps countries evaluate and strengthen primary healthcare performance through standardized indicators and evidence-based tools. It improves monitoring and evidence-informed policy decisions [26,27].

International organizations continue to work with GCC countries to strengthen research capacity and academic activity in the health sector [28]. These collaborations also support the development of new models of care and broader capacity building. In addition, the World Health Organization has partnered with WONCA, the World Organization of National Colleges, Academies and Academic Associations of General Practitioners/Family Physicians, to support the expansion of family medicine programs, particularly in resource-limited settings [29]. This partnership represents an important step in strengthening service delivery and improving the organization of primary healthcare.

As demand for primary healthcare continues to grow across the region, the need for family medicine training is also increasing at both national and international levels [30]. Cross-border training partnerships are especially valuable in helping countries respond to these rising service demands. Capacity development also depends heavily on how effectively human resources are deployed across GCC health systems [31]. In support of this, the UAE, Qatar, and other GCC countries have established formal links with universities in North America and Europe [32]. These partnerships are intended to help train healthcare professionals with the knowledge and skills needed for family medicine practice. A growing body of evidence highlights the critical role of international collaboration and capacity-building initiatives in strengthening healthcare systems [28,31].

Academic networks within GCC countries, together with WHO collaborating centers, continue to support ongoing international research initiatives [5,33]. The broader objective of these efforts is to refine existing healthcare delivery mechanisms through the integration of robust evidence and effective innovations. Recent initiatives across the GCC also reflect a strong commitment engagement, the improvement of mental health services, and the more systematic management of non-communicable diseases.

Long-term investment in primary healthcare remains essential for the development of health systems that are capable of sustaining continuity of care while adapting to pressures such as pandemics and population ageing. GCC countries have already directed substantial financial resources towards this sector [34]. Future investment should continue to prioritize areas with the greatest public health relevance, particularly non-communicable diseases, mental health, and community engagement [26]. Ultimately, the strength of primary healthcare systems depends not on funding alone, but on the extent to which political commitment, sound policy, effective collaboration, and sustained financial support are brought together [5,19,26].

## 4. Medical Education and Training

### 4.1. Historical Context and Current Landscape

Saudi Arabia was the first GCC country to establish formal medical education, with its first medical school opening in 1969. Bahrain, Kuwait, and the UAE followed at later point [35,36,37]. In Qatar, medical education was introduced into the university curriculum in 2002 [38]. Together, these developments reflected a broader regional expansion in medical training and were accompanied by the growth of nearly 40 universities involved in medical education across GCC countries [39]. During the same period, countries in the region also moved towards closer alignment with international accreditation standards and increased the availability of postgraduate medical training [40].

Several postgraduate family medicine programs have since gained accreditation from the Accreditation Council for Graduate Medical Education International [41]. In Kuwait, some academic institutions have incorporated elements of the educational model used by the Royal College of General Practitioners in the United Kingdom [42]. In Saudi Arabia, a number of postgraduate family medicine programs have been shaped by Canadian competency-based medical education models [43,44]. Taken together, these developments reflect a regional willingness to draw on well-established international approaches while adapting them to local contexts. Nevertheless, medical education across the GCC still lacks a sufficiently coherent pedagogical framework, which continues to affect the overall standards and consistency of training in the region [39,40].

### 4.2. Family Medicine Residency

There has been a growing need to establish and expand family medicine training programs in response to evolving healthcare demands [45]. Such programs are essential to meeting changing patient care needs and equipping healthcare providers with the necessary clinical competencies. Training interventions should also be tailored to strengthen leadership, quality improvement, administrative, and resource management skills among family physicians [31,45,46]. The introduction of a health systems management curriculum by the Family Medicine Department of the Oman Medical Specialty Broad was intended to improve the resource management capacity of medical professionals [46]. In 2015, this specialty program received ACGME-I accreditation [47]. However, efforts to expand training programs have been constrained by high clinical workload, low trainee enrolment, and an insufficient number of accredited training sites [14,31,45].

### 4.3. Continuing Professional Development

GCC countries have sought to strengthen family medicine training through a learner-centered approach [37]. To address the limited availability of educators, some programs have drawn on adult learning theory by enabling the same trainers to contribute to both competency assessment and learning development [48,49]. Research has also indicated considerable potential for expanding e-learning platforms in training delivery, given their scalability and acceptability among trainees in the GCC [50]. For example, Tebyan is an e-learning platform implemented by the Oman Medical Specialty Board [51]. Its primary purpose is to enhance collaboration among participants involved in training and medical education. It also contributes to continuing professional development in family medicine across the GCC. To remain effective, continuing professional development programs should be aligned with international standards in medical education while also reflecting changes in workforce demands, community health priorities, and the expanding digital context of healthcare delivery [31].

### 4.4. Interprofessional Education

Although interprofessional education remains at an early stage in many GCC countries, the UAE and Qatar have made notable progress through the development of stronger institutional frameworks and dedicated working committees [52,53]. Across the region, there is increasing recognition of the need to strengthen interprofessional education through improved accreditation processes and better alignment of national frameworks. Evidence has shown that interprofessional education can improve care coordination, patient safety, and team-based clinical outcomes [54,55]. There are examples of interprofessional education initiatives in GCC countries, especially in the UAE and Qatar. These countries have implemented structured programs developed within academic and healthcare institutions. These initiatives focus on the use of collaborative training sessions to promote interprofessional collaboration. The approach is meant to improve communication and shared decision-making in the clinical setting. Early experiences show that successful implementation depends on strong institutional support, facility training, and alignment with accreditation and curricular frameworks. Lessons from these programs are the importance of integrating interprofessional education in early stages of training, promoting a culture of collaboration, and ensuring that educational activities are closely linked to clinical practice. These approaches have the potential to improve care coordination, patient safety, and overall healthcare outcomes in the GCC context [52,53,54,55].

## 5. Digital Transformation

### 5.1. Digital Health

The integration of telemedicine, Cerner- and Epic-type electronic medical record systems, and artificial intelligence has continued to expand the scope and clinical application of family medicine [56,57,58,59,60]. The use of technological innovation in family medicine can improve the quality of patient care by supporting healthcare providers and empowering patients through greater health awareness, improved access to services, and more informed shared decision-making [61,62]. The increased use of artificial intelligence applications may also enhance patient awareness of health and self-care by improving understanding of personal health concerns and their underlying causes. More broadly, the role of technology in family medicine is to strengthen communication between patients and physicians, support the effective use of self-management strategies, and improve the flow of healthcare information [63,64].

### 5.2. Artificial Intelligence and Medical Decision-Making

Technological support can reduce physicians’ workload, allowing more time for the assessment of patient information and helping to lower the risk of diagnostic and treatment errors [65]. Pavuluri et al. [65] explored the role of AI in addressing healthcare workforce challenges, potential to reduce clinician burnout through workflow optimization, and decision support tools. Basu et al. [66], in a population health-focused study, examined the application of AI in primary care settings and emphasized its role in improving risk stratification and preventive care strategies at the population level. Mutharasan and Walradt [67] provided a comprehensive overview of the use of AI in population health. The study demonstrates potential of AI to enhance disease prediction, early detection, and health system planning. These studies support the growing role of AI in strengthening clinical decision-making and healthcare system performance. It may further reduce cognitive burden and improve physicians’ adherence to clinical practice guidelines. However, concerns have been raised regarding the potential impact of artificial intelligence on person-centered care, particularly when healthcare delivery becomes increasingly automated. Addressing this concern requires the development of appropriate regulatory guidance and patient support frameworks at both national and international levels [6,63,67].

### 5.3. Care Continuity Improvement with Electronic Health Records

Many of the limitations associated with paper-based documentation have been addressed through the adoption of electronic health records [68,69]. Their systemic use has improved healthcare providers’ access to relevant patient information. The implementation of advanced electronic medical records has also contributed to substantial improvements in population health management and continuity of care [70]. The incorporation of analytical functions within electronic health records, together with e-prescribing systems, helps physicians identify potential drug–drug interactions, recognize contraindications, and determine appropriate medication dosages, thereby reducing prescribing errors [71]. These systems also support more consistent collaboration among physicians, pharmacists, patients, and caregivers. In addition, e-prescribing can help estimate disease prevalence and patterns of drug misuse, while enabling the monitoring of commonly used therapies through medication-use data. Electronic appointment systems have further improved scheduling processes [72,73,74]. Moreover, the automation of physician dictation and transcription has contributed to greater accuracy in medical records [75].

### 5.4. Telemedicine in Family Medicine

The introduction of telemedicine into family medicine practice has reduced the need for face-to-face consultations [76]. It has also improved access to care and supported service delivery in resource-limited settings. Telemedicine has increasingly been adopted in primary care and family medicine setting across GCC. The application of this technology has significantly increased after the COVID-19 pandemic. Evidence from the region supports the use of telemedicine in healthcare delivery. An example is a cross-sectional study conducted in Oman involving primary healthcare providers (*n* ≈ 143) demonstrated the feasibility and moderate usability of telemedicine services. These findings show the potential of the technology to support service delivery in areas requiring further improvement [77]. A cross-sectional study from Saudi Arabia involving patients attending primary care services (*n* = 377) reported high levels of satisfaction with telemedicine, particularly in terms of accessibility and convenience [78]. In the United Arab Emirates, a cross-sectional study involving patients using telemedicine services (*n* = 515) found high patient satisfaction and improved access to care in both hospital and community-based settings [79]. These studies demonstrate that telemedicine is increasingly becoming integrated into primary care settings across GCC and it offers significant benefits in accessibility, continuity of care, and patient engagement. However, major limitations of telemedicine-based care include low levels of digital literacy and concerns related to data protection [80]. Although digital transformation has reshaped family medicine practice, it continues to face challenges in protecting patient choices and maintaining equitable, value-based care across GCC countries [81].

## 6. Future Opportunities for Research and Innovation in Family Medicine in the GCC Countries

Research continues to play a central role in the development of effective and sustainable healthcare systems by informing clinical practice, supporting innovation, and guiding health policy [82,83]. In countries with established healthcare systems, research has long been seen as a core element of health sector development, and sustained investment in research capacity and scholarly output remains an important priority. In the GCC, however, progress in research has been less pronounced than the advances made in healthcare infrastructure and population health. Although substantial investment has been made and major health indicators have improved, research productivity remains comparatively limited. A regional bibliometric analysis by Al-Farsi et al. found that GCC countries had lower publication rates and weaker citation impact than North America and Western Europe, underscoring a persistent gap between progress in healthcare delivery and the generation of new knowledge [84].

These challenges are even more apparent in family medicine. Globally, research in family medicine has historically lagged behind that of other specialties in both funding and productivity [85]. Evidence from the United States indicates that fewer than one-quarter of family medicine departments maintain sustainable research programs, and that the specialty receives only a small proportion of national research funding [86]. For decades, family medicine has had to define its academic identity alongside organ-based and disease-focused disciplines, a struggle that has directly affected its research productivity [85]. As a result, family medicine research output worldwide continues to trail that of most other medical specialties.

In the GCC, barriers to research in family medicine arise from both specialty-specific challenges and wider systemic constraints. Family medicine is still a relatively young discipline in the region, and public understanding of its central role in effective health systems remains limited [4,14,85]. At the policy level, decision-makers have traditionally prioritized investment in tertiary hospitals and subspecialty services, often at the expense of primary care research and development. The perceived lower prestige of family medicine, which is largely delivered in health centers with basic infrastructure and essential equipment, has further contributed to its undervaluation in policy agendas. These difficulties are compounded by structural barriers, including limited research infrastructure, lack of protected time, shortages of trained researchers, and inadequate funding.

Regional evidence suggests that these barriers do not reflect a lack of interest or motivation. A study from Saudi Arabia showed that family physicians value research and recognize its importance, yet face substantial challenges related to time, mentorship, funding, and training in biostatistics and scientific writing [87]. These findings closely mirror international experiences and support the view that the limitations affecting family medicine research are systemic rather than individual [83,86].

The accelerating digital transformation across GCC healthcare systems offers an important opportunity to advance family medicine research. Electronic medical records, national data platforms, and artificial intelligence applications provide a strong foundation for population-based research, and quality improvement initiatives. Family physicians are well positioned to lead this work and translate these data into improvements in clinical practice and healthcare systems.

Experience from high-income countries shows that progress is achievable. Studies have demonstrated that relatively simple, low-cost interventions, such as research skills workshops, structured mentorship, and formal recognition of scholarly activity, can shift departmental cultures from a predominantly service-oriented model to a more productive academic environment [88]. More broadly, the growth of family medicine research has been supported by institutional leadership, policy advocacy, and the deliberate integration of research with clinical improvement [89]. A recent scoping review also noted that lasting progress in family medicine research depends on several interconnected elements, particularly training, opportunities, mentorship, professional networking, and dedicated funding support [90].

Future research in family medicine across GCC should focus on areas that generate actionable evidence for health system strengthening and policy development. It is crucial to use robust studies evaluating the impact of family medicine on preventive care, early disease detection, and management of non-communicable diseases in the region. Research focusing on cost-effectiveness, reduction in hospital admissions, and improved patient outcomes will demonstrate the value of investing in primary care. Implementing research addressing the effectiveness of patient-centered care models such as Patient-Centered Medical Home and integration of digital health solutions can provide practical guidance for scaling up successful interventions. Use of research focusing on health equity, access to care, and population health outcomes will help address disparities within GCC countries. Generating locally relevant evidence evaluating economic and clinical benefits of family medicine will inform policy decisions and strengthen political commitment toward sustained investment in primary healthcare systems.

## 7. Public Collaboration

### 7.1. Multidisciplinary Procedures

Integrating family medicine into existing healthcare models is essential for strengthening public engagement and promoting person-centered care [91,92]. The effectiveness of multidisciplinary healthcare approaches depends on sustained and trustworthy collaborations among communities, patients, and healthcare professionals [93]. Recent developments in family medicine are reflected in the Patient-Centered Medical Home (PCMH) model, which has been used to improve several aspects of care, including lifestyle modification, specialist referral pathways, management of non-communicable diseases, and preventive care [94,95]. Evidence also supports the value of interprofessional collaboration in improving the management of chronic diseases [96].

### 7.2. Medical Tourism

Medical tourism hubs in GCC countries draw on strategic geographic location, technological advancement, and specialized services to improve the patient care experience [97,98]. The development of family medicine in the GCC plays a complementary rather than central role in medical tourism, primarily through continuity of care, pre-travel assessment, and post-treatment follow-up [99]. Individualized family medicine approaches are intended to tailor care plans to patients’ specific needs [100].

### 7.3. Preventive Health and Promotion Campaigns

Preventive health strategies aim to improve population health while reducing long-term treatment costs [101]. Population-level campaigns led by health ministries across GCC countries use community partnerships and digital platforms to strengthen health literacy, promote vaccination uptake, and raise awareness of risk factors for non-communicable diseases, including metabolic, cardiovascular, and mental health conditions [7,21,102].

Family physicians in integrated healthcare systems play a role as gatekeepers, care coordinators, and health advocates. They are responsible for ensuring continuity of care across different levels of the healthcare system. The position allows the professionals to facilitate effective multidisciplinary collaboration, improve care integration, and promote patient-centered decision-making. Specialized tertiary services significantly influence medical tourism in the GCC. The role of family medicine in this care delivery process is to ensure continuity and patient safety. Family physicians are responsible for pre-travel risk assessment, management of chronic conditions before traveling, and post-treatment after patients return to their home countries [99,100]. The supportive role helps maintain continuity of care and prevents complications. Family medicine plays an important role in preventive healthcare. It improves healthcare outcomes, reduces hospitalizations, and lowers the cost of care. Longitudinal patient relationships allow family physicians to implement screening programs, deliver lifestyle interventions, and support early detection and management of non-communicable diseases. The prevention function represents one of the most effective and sustainable strategies for improving population health and reducing long-term health burden [7,21,102].

Non-governmental organizations (NGOs), professional bodies, and regional medical associations play a critical role in strengthening public collaboration in the GCC. Medical societies and syndicates contribute to continuing professional development, dissemination of clinical guidelines, and the promotion of best practices in family medicine. These organizations support research networks, organize scientific meetings, and facilitate knowledge exchange across countries. NGOs and community-based organizations are key partners in health promotion and disease prevention initiatives, particularly in areas such as non-communicable diseases, mental health awareness, and vaccination campaigns. Collaboration at the regional level occurs through professional networks and organizations. These organizations harmonize standards, build capacity, and share expertise across GCC countries. It is important to further strengthen engagement with these stakeholders to enhance community participation, improve health literacy, and support the development of more integrated and patient-centered primary healthcare systems [5,29].

## 8. Recommendations

Future research in family medicine across Gulf Cooperation Council (GCC) countries should move toward rigorous, system-oriented, and impact driven studies. Multicenter research can evaluate effectiveness of primary care interventions on patient outcomes, healthcare utilization, and cost-efficiency within the regional context. Longitudinal and implementation research designs will evaluate real world impact of national strategies such as Vision 2030 and Vision 2040. It will provide a comprehensive understanding on strengthening primary care systems. It is necessary for future research to focus on the development and evaluation of context-specific models of care. Key areas include patient-centered medical homes, integrated multidisciplinary care, and digitally enabled primary care services.

It is important to evaluate expansion of practice-based research networks across GCC countries. The information will help generate locally relevant evidence that reflects realities of primary care delivery. Strengthening research capacity improves sustainable academic productivity in family medicine. Approaches to achieve the goal include the use of structured mentorship programs, protected research time, and dedicated funding mechanisms. Future studies should evaluate the role of emerging technologies in the healthcare setting. There has been the emergence of artificial intelligence and big data analytics that are expected to improve decision-making, population health management, and health system planning. Qualitative and mixed-methods research is helpful in understanding patient experiences, healthcare provider perspectives, and barriers to care within diverse GCC populations.

Regional collaboration is critical for data sharing, standardization of methodologies, and coordinated research agendas. This approach can involve establishing GCC-wide research framework or consortium for family medicine. These efforts will help advance the academic integrity of family medicine and ensure that future healthcare policies and practices in the GCC are founded on high-quality evidence.

## 9. Limitations

This review has several limitations. Most of the available evidence from GCC countries is derived from cross-sectional and descriptive studies, which limit causal inferences and the assessment of long-term outcomes. In addition, the quantity and quality of published research vary considerably across GCC countries, resulting in regional heterogeneity in the available evidence. Findings related to emerging innovations, such as telemedicine and artificial intelligence, should therefore be interpreted with caution, as evidence regarding their long-term effectiveness and sustainability remains limited. Future research should prioritize longitudinal, interventional, and implementation studies to strengthen the evidence base for family medicine and primary healthcare in the region.

## 10. Conclusions

The adoption of innovative multidisciplinary approaches represents an important step in strengthening and advancing family medicine practice across GCC countries. Further progress will depend on stronger international collaboration, supportive healthcare policies, technological innovation, and continued development of medical education and training. There is a clear need for resilient, patient-centered healthcare systems in the GCC that incorporate global best practices while strengthening the capacity of the healthcare workforce. Greater integration of family medicine within mainstream healthcare systems is also needed to improve the prevention and long-term management of non-communicable diseases. Ultimately, stronger community engagement and the development of more accountable and innovative healthcare systems across GCC countries will require sustained investment in family medicine. Findings of this review are primarily situated within the GCC context. The majority of identified challenges and opportunities are relevant in other regions undergoing rapid health system transformation. The findings can be applied in countries with a high burden of non-communicable diseases, evolving healthcare infrastructures, and growing demands of cost-effective care.

## Figures and Tables

**Table 1:** A summary of key national health strategies and their primary focus areas across GCC countries.

Country	Key Policy/Strategy	Focus Area
Oman	Health Vision 2040	Strengthening primary healthcare, universal health coverage, digital transformation, workforce development, and prevention of non-communicable diseases
Saudi Arabia	Vision 2030	Primary healthcare transformation, prevention, integrated care, privatization, and digital health
UAE	National Health Strategy	Digital health innovation, healthcare accessibility, quality improvement, and patient-centered care
Qatar	National Health Strategy	Integrated care, population health management, prevention, and healthcare quality
Bahrain	National Health Plan	Expansion of primary care services, universal access, prevention, and service quality improvement
Kuwait	MOH Strategic Plan	Workforce development, service delivery improvement, healthcare infrastructure, and digital transformation

## Data Availability

No new data were created or analyzed in this study. Data sharing is not applicable to this article.
